# Mycetoma: A Long Journey from Neglect

**DOI:** 10.1371/journal.pntd.0004244

**Published:** 2016-01-21

**Authors:** Eduard E. Zijlstra, Wendy W. J. van de Sande, Ahmed H. Fahal

**Affiliations:** 1 Rotterdam Centre for Tropical Medicine, Rotterdam, The Netherlands; 2 Erasmus University Medical Center, Department of Medical Microbiology and Infectious Diseases, Rotterdam, The Netherlands; 3 Mycetoma Research Center, Soba University Hospital, Khartoum, Sudan; University of Tennessee, UNITED STATES

Even in the tropical medicine community, mentioning mycetoma often raises eyebrows and requires further explanation. This condition has all the ingredients of neglect: it affects the poorest segment of the population in remote areas, the course of the disease is slow and chronic, and health services in the endemic areas do not have trained staff, adequate diagnostic tools, or treatment. There is uncertainty about the route of transmission, which contributes to lack of effective national control programs. The associated stigma has severe socioeconomic consequences: children drop out of school and their peer group, and young adults cannot finish their training or find a job or a partner. Patients are affected psychologically because of the lack of health services, the physical disability, and the lack of prospects, as the outcome of treatment is poor and often leads to amputation of the affected part [1].

Although mycetoma affects many countries in each part of the (sub)tropics, the disease burden is not well-known [2]. Mycetoma may be caused by bacteria (actinomycetoma), in particular *Nocardia* spp., or by fungi (eumycetoma), of which *Madurella mycetomatis* is the most common. It is thought that the microorganisms enter the skin through a thorn prick or other breach of the skin, after which the typical subcutaneous mass develops, usually on the foot. Swelling, sinus formation, and discharge of grains are considered characteristic of the disease. Although the foot is most commonly affected, all parts of the body may be involved, either directly or through lymphatic or hematogenous spread that may include the spinal cord and the brain.

Diagnosis in the field is usually clinical; there is no point-of-care diagnostic test. In referral centers, ultrasound, magnetic resonance imaging (MRI), and fine needle aspiration or biopsy are used for accurate description of the extent of the lesion and the causative microorganism [3].

The key neglect is in treatment; while repeated antibiotic courses with amikacin and cotrimoxazole are used in actinomycetoma with good result (cure rate >90%), this is not the case for eumycetoma. Ketoconazole, which was used previously in many countries, has been banned by the Food and Drug Administration (FDA) and the European Medicines Agency (EMA) because of toxicity, with its use restricted to certain indications. Currently, only itraconazole is used; the treatment duration is long, with a mean of 12 months, after which the remaining lesion is removed surgically. Not uncommonly, the fungus can still be cultured from the surgical specimen, which explains the low cure rate (26%); 55% of patients do not complete the treatment, often because they cannot afford the drug [4]. Recurrence is therefore common and may lead to amputation. The choice of drugs is extremely limited, as only azoles are used: all other classes of antifungals are ineffective in vitro. The issue of treatment is further compounded by concomitant secondary bacterial infection, the variability in the extent and severity of the lesion, and the presence of bone involvement.

In 2013, the Mycetoma Consortium was founded by a number of experts, and research priorities were listed [5]. The Consortium has promoted advocacy that has resulted in the inclusion of mycetoma on the WHO list of neglected diseases, albeit under “other conditions.” While this effort has attracted international attention in the media, it is not sufficient to raise donors’ interest, and a proposal to include mycetoma in the “top 17” list of neglected tropical diseases (NTDs) will be submitted to the executive board of WHO to be discussed in the World Health Assembly in 2016. Symposia were organized during various scientific conferences in the United States and Australia and in the European Conference of Tropical Medicine and Hygiene (ECTMIH) in Basel, September 2015. *PLOS NTDs* graciously agreed to this “Mycetoma Collection,” and a series of papers have been submitted and published. Documentaries have been produced by WHO and Aljazeera. The Mycetoma Research Center in Khartoum, Sudan, has recently been recognized as a WHO Collaborative Center for mycetoma.

A potential breakthrough in treatment of eumycetoma could be fosravuconazole (formerly known as E1224), which is produced by Eisai, Japan. This is a prodrug of ravuconazole and was developed as an antifungal; it has also been studied in Chagas disease by the Drugs for Neglected Diseases initiative (DNDi). Being an azole, its efficacy was tested in vitro against *M*. *mycetomatis*, and it showed excellent sensitivity [6]. A proof-of-concept trial in eumycetoma patients with limited lesions is planned, comparing two dosages of fosravuconazole with standard treatment of itraconazole. This study results from a partnership between DNDi and Eisai and will be carried out in Sudan.

While this will be the first randomized study of its kind in eumycetoma, more work is needed. If successful, fosravuconazole needs to be studied in more complex eumycetoma patients. Worryingly, after fosravuconazole there is no back-up: there is no pipeline for new compounds, and eumycetoma may become virtually untreatable, with amputation as the only option.

Other areas in research also need to be addressed urgently. Obviously, the burden of disease needs to be described. A point-of-care test that ideally also could be used as a biomarker to monitor treatment is needed for diagnosis in the field. The route of transmission needs to be clarified to identify potential methods for intervention. This should include the use of protective footwear, which was was found to be associated with lower odds for a number of neglected tropical diseases (e.g., Buruli ulcer, strongyloides, and soil-transmitted helminths); however, there are no data for mycetoma [7]. The role of coinfection in mounting immune responses needs to be elucidated.

The most important impact would, however, come from increased international recognition by governments, WHO, nongovernmental organizations (NGOs), and donors. For the short term, a mycetoma management model that would include mobile surgical teams visiting the endemic areas has been proposed [8]. For the long term, national control programs need to be established, with the upgrading of health services in the endemic areas—by training staff and providing adequate tools for diagnosis and treatment—as the main priority.
